# Supplementary material for: Magnetic Anisotropy and Field-induced Slow Relaxation of Magnetization in Tetracoordinate CoII Compound [Co(CH3-im)2Cl2]
Source: Materials (Basel). 2017 Feb 28;10(3):249. doi: 10.3390/ma10030249 (PMC5503352; doi:10.3390/ma10030249)
Supplement: Supplementary file 1 [file materials-10-00249-s001.pdf]

Supplementary Material: Magnetic Anisotropy and Field-induced Slow Relaxation of Magnetization in Tetracoordinate Co^{II} Compound $[Co(CH_3\text{-im})_2Cl_2]$

Ivan Nemec, Radovan Herchel, Michal Kern, Petr Neugebauer, Joris van Slageren, and Zdeněk Trávníček *

Contents

Figure S1. X-ray powder diffraction pattern for 1. .... S2

Figure S2. HFEPR frequency dependence of 1 at 8 K........ S2

Figure S3. HFEPR frequency dependence of 1 at 13 K........ S3

Figure S4. HFEPR frequency dependence of 1 at 22 K........ S3

Figure S5. HFEPR temperature dependence of 1 at 260 GHz. S4

Figure S6. HFEPR temperature dependence of 1 at 300 GHz S4

Figure S7. HFEPR temperature dependence of 1 at 340 GHz S5

Figure S8. HFEPR temperature dependence of 1 at 380 GHz S5

Figure S9. HFEPR frequency dependence of 1 at 8 K in the range from 100 to 500 GHz S6

Figure S10. HFEPR detail of variable temperature measurements S6

Figure S11. Frequency/Field plot for the HFEPR measurements and calculations........ S7

Figure S12. The in-phase χ_{real} and out-of-phase χ_{imag} molar susceptibilities for 1 .... S8

Figure S13. Analysis of in-phase $_{real}$ and out-of-phase $_{imag}$ molar susceptibilities for 1........ S9

Table S1. Parameters of one-component Debye model for 1........ S9

Table S2. Individual contributions to D-tensor of 1 calculated by CASSCF/NEVPT2........ S10

Table S3. Energy levels (cm-1) of ligand field multiplets in zero magnetic field S11

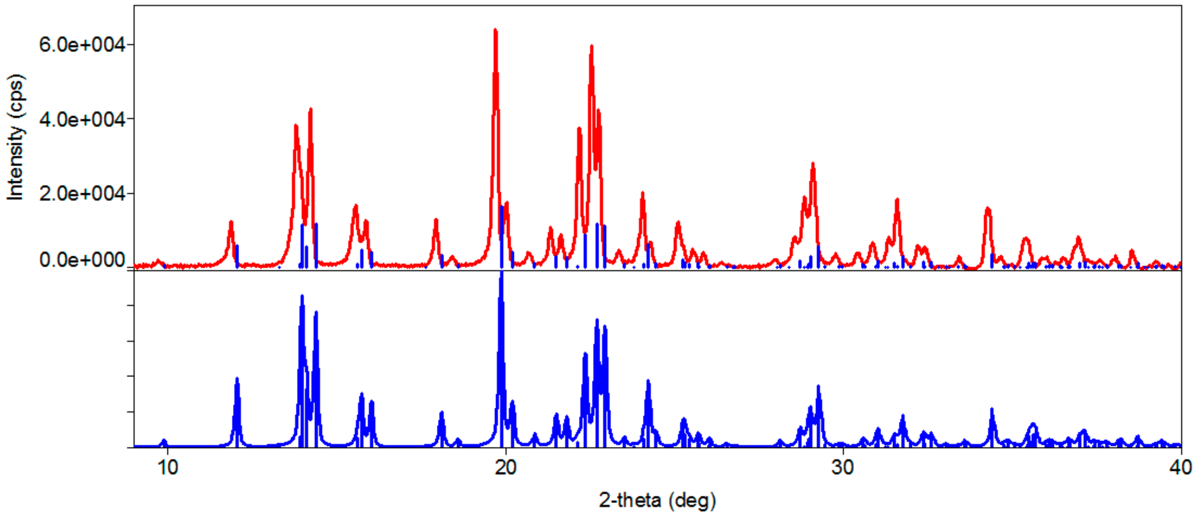

Figure S1. X-ray powder diffraction pattern for 1. Experimental data are shown as a red line, calculated as a blue line.

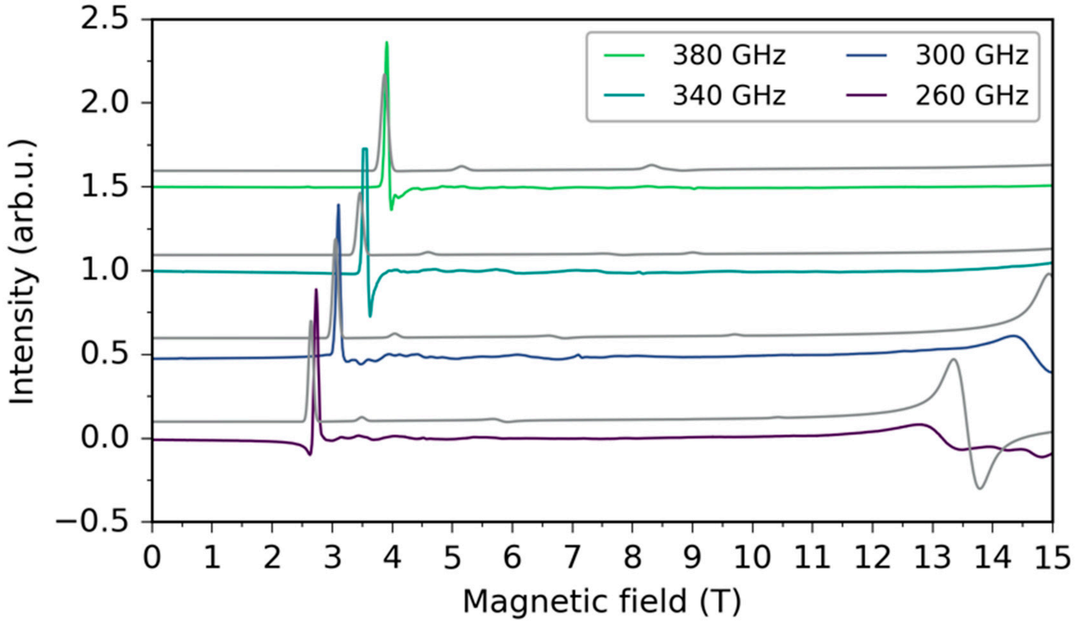

Figure S2. HFEPR frequency dependence of 1 at 8 K. The colored lines represent experimental data, while the grey lines calculated ones.

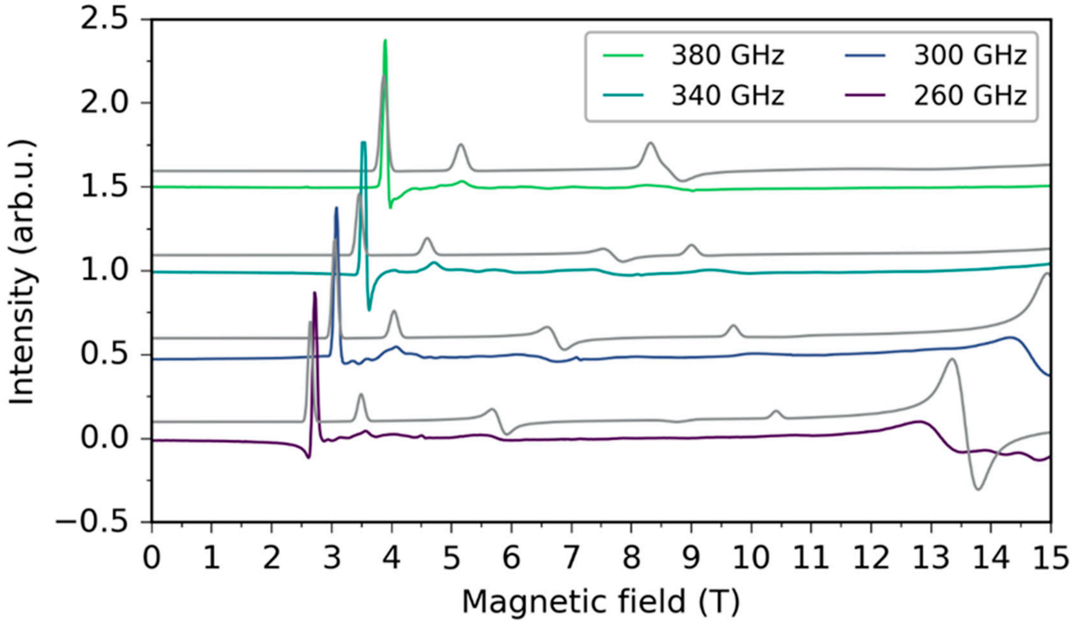

Figure S3. HFEPR frequency dependence of 1 at 13 K. The colored lines represent experimental data, while the grey lines calculated ones.

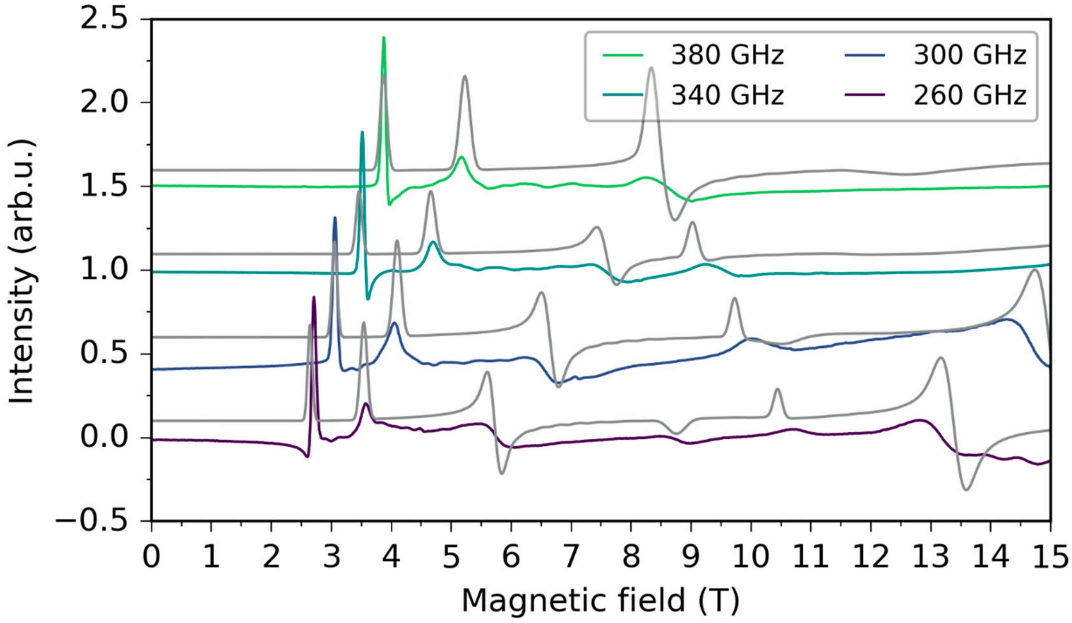

Figure S4. HFEPR frequency dependence of 1 at 22 K. The colored lines represent experimental data, while the grey lines calculated ones.

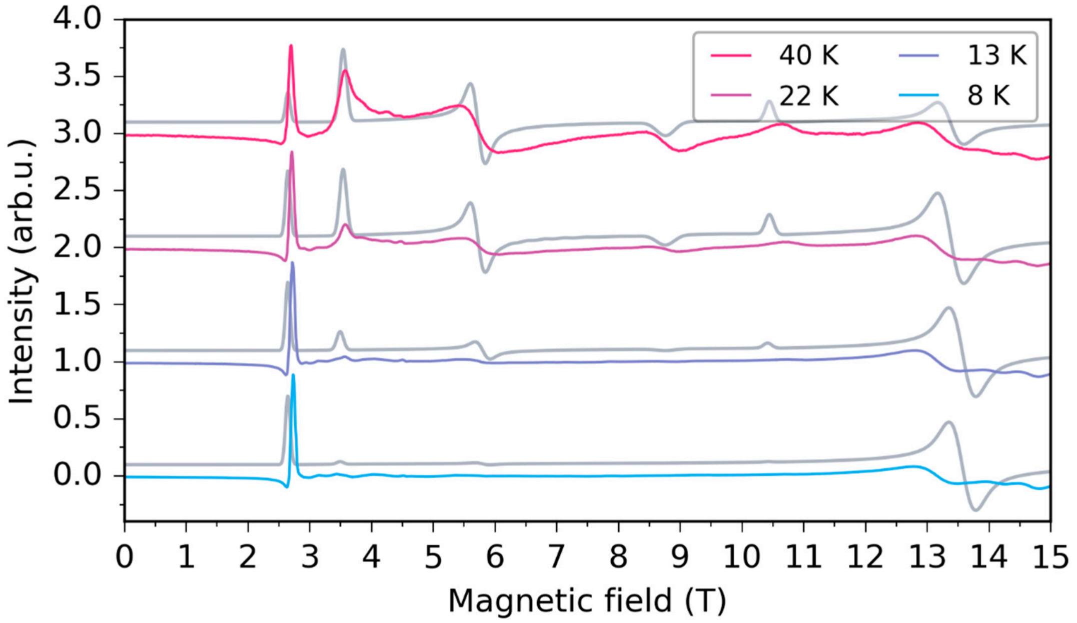

Figure S5. HFEPR temperature dependence of 1 at 260 GHz. The colored lines represent experimental data, while the grey lines calculated ones.

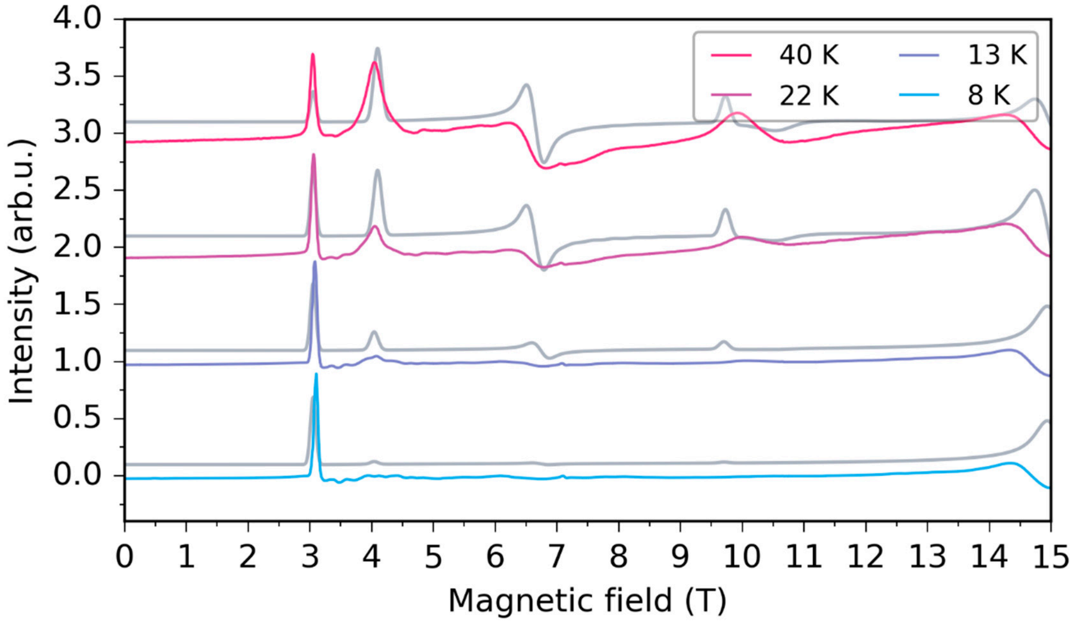

Figure S6. HFEPR temperature dependence of 1 at 300 GHz. The colored lines represent experimental data, while the grey lines calculated ones.

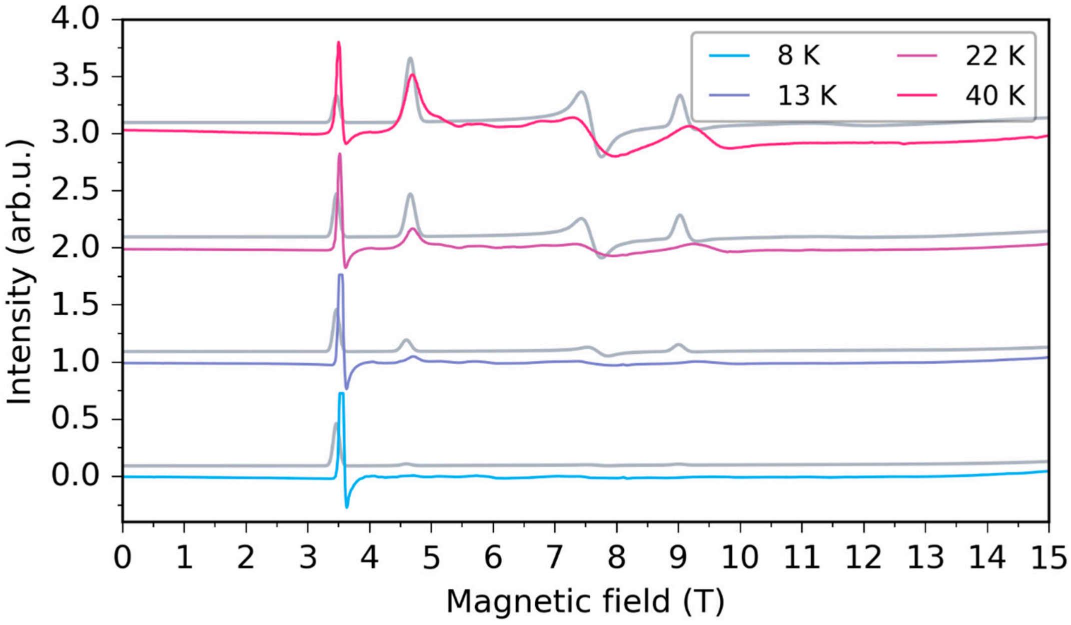

Figure S7. HFEPR temperature dependence of 1 at 340 GHz. The colored lines represent experimental data, while the grey lines calculated ones.

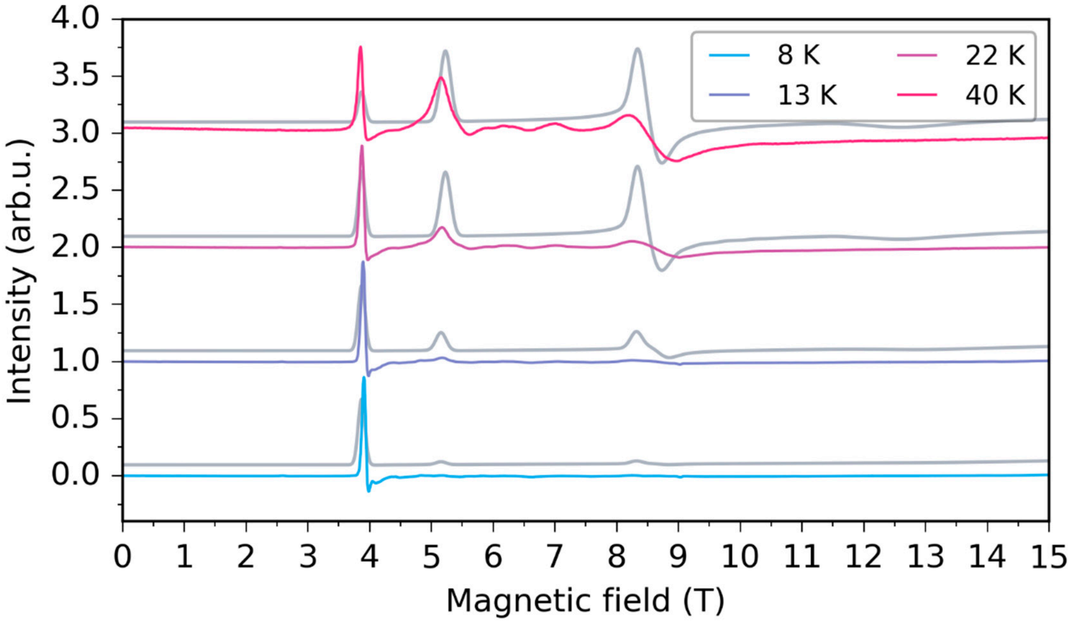

Figure S8. HFEPR temperature dependence of 1 at 380 GHz. The colored lines represent experimental data, while the grey lines calculated ones.

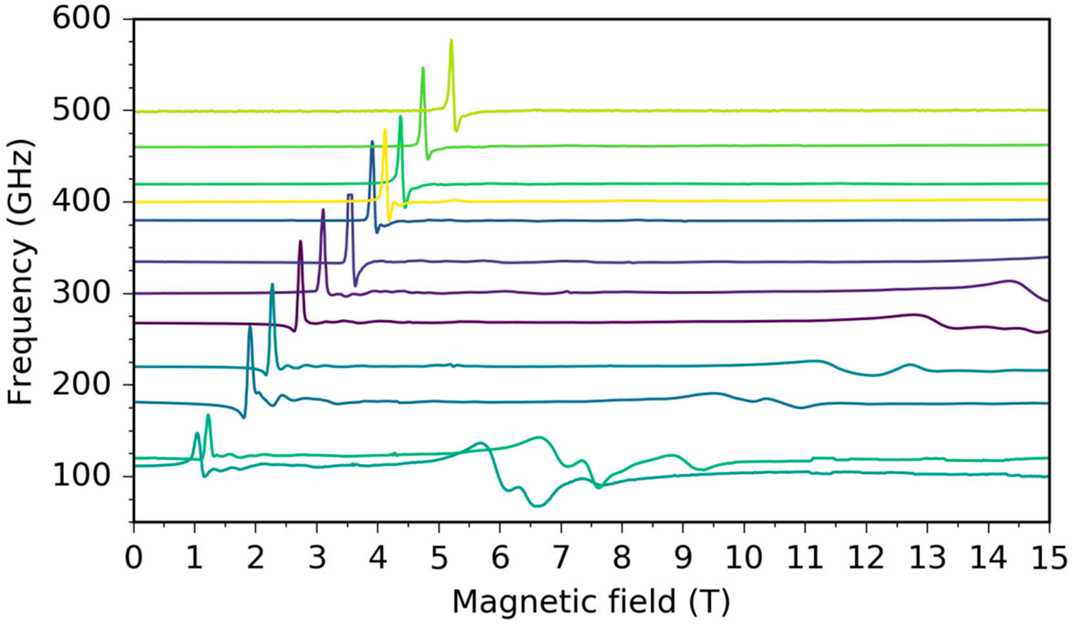

Figure S9. HFEPR frequency dependence of 1 at 8 K in the range from 100 to 500 GHz.

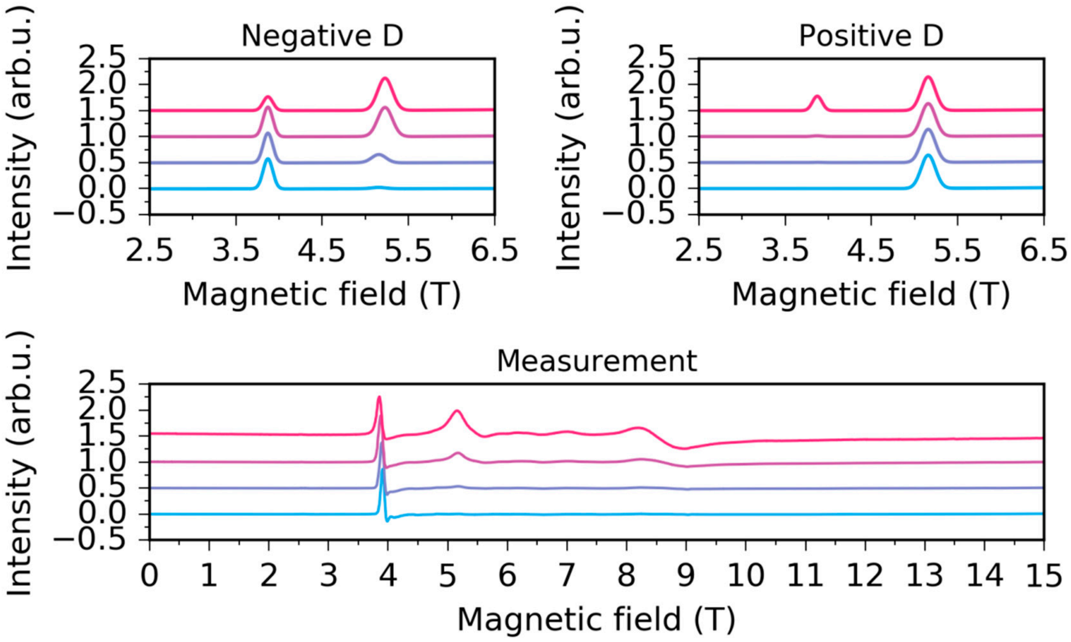

Figure S10. HFEPR detail of variable temperature measurements and calculations at 380 GHz. The sign of the ZFS parameter D can be best distinguished by focusing on the relative intensities of the doublet between 3 and 6 T.

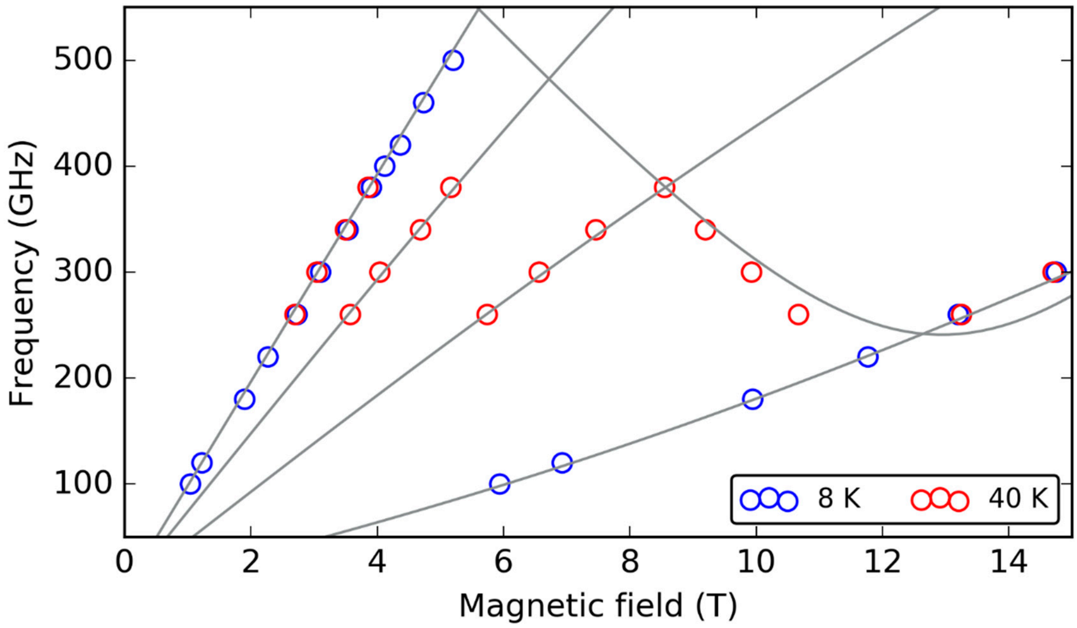

Figure S11. Frequency/Field plot for the HFEPR measurements and calculations. The dots represent the dominant, confidently assignable peaks along with temperature at which they were identified, while grey lines represent calculated resonance frequencies. The calculated data were obtained by using the EasySpin *resfreqs_matrix* function for *x*, *y* and *z* directions and subsequently only the transitions observed in the measured powder spectrum were picked.

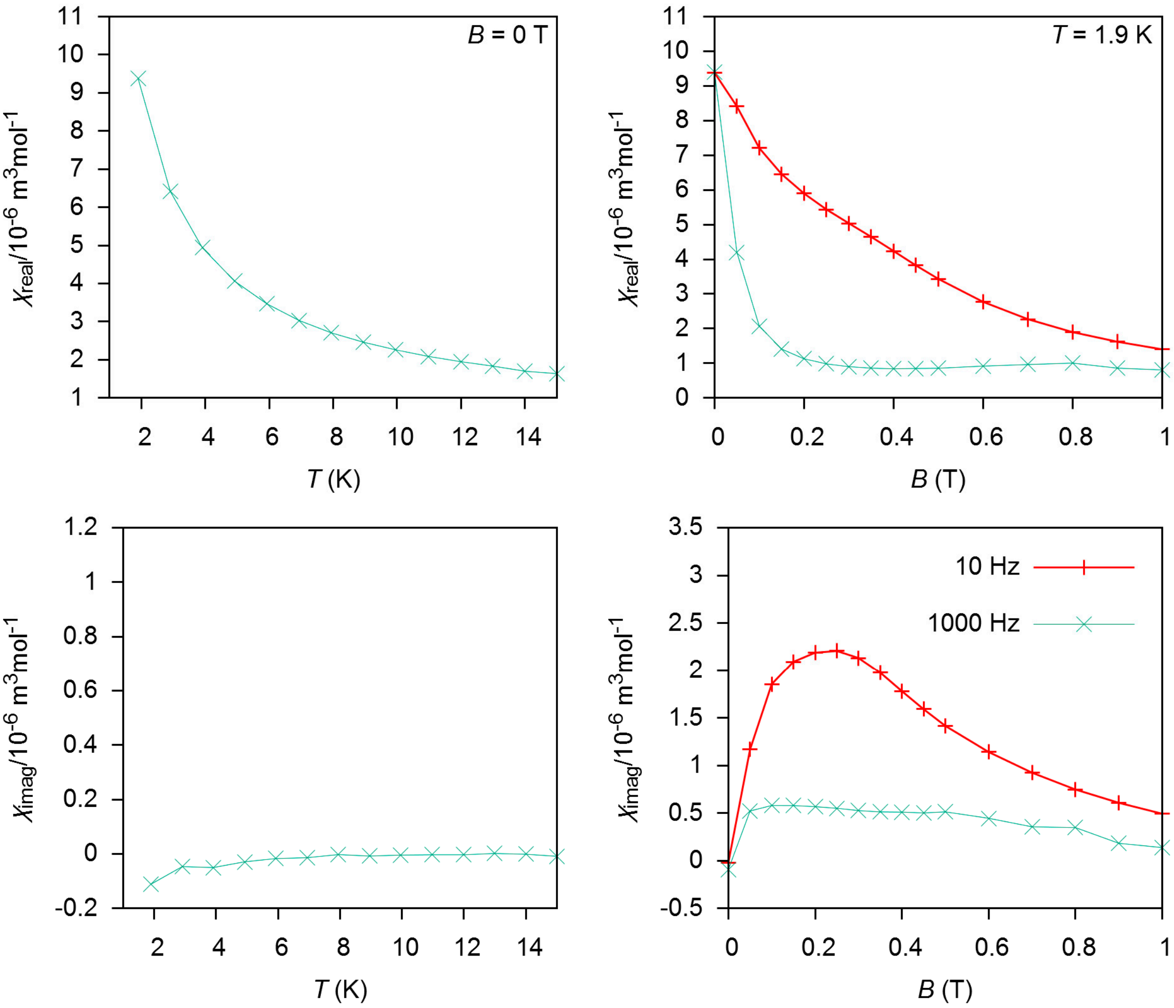

Figure S12. The in-phase χ_{real} and out-of-phase χ_{imag} molar susceptibilities for 1 at zero static magnetic field (left) and in non-zero static field (right). The lines serve as guides for the eyes.

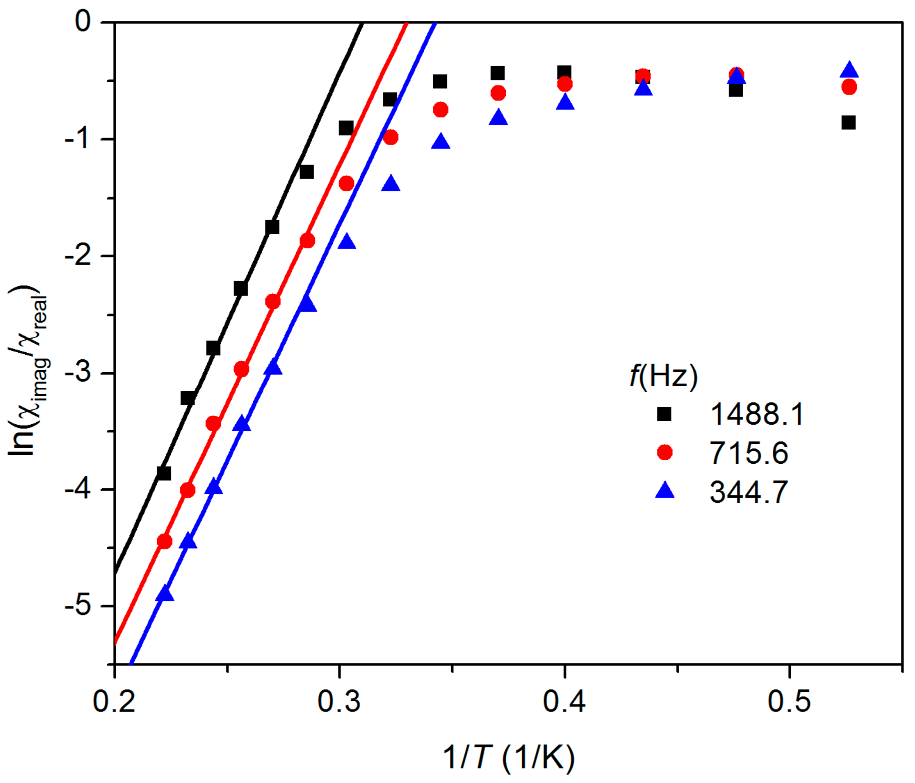

Figure S13. Analysis of in-phase $_{\mathrm{real}}$ and out-of-phase $_{\mathrm{imag}}$ molar susceptibilities for 1 measured at the applied external field B_{dc} = 0.2 T according to Equation (4). Full points – experimental data, full lines – calculated data with parameters listed in the text.

Table S1. Parameters of one-component Debye model for 1 derived according Equation (3) in main text.

T/K	$_{\mathrm{S}}$/(10^{-6} m^{3}mol^{-1})	$_{\mathrm{T}}$/(10^{-6} m^{3}mol^{-1})		/(10^{-3} s)
1.9	0.545	9.938	0.440	10.757
2.1	0.568	8.818	0.451	5.422
2.3	0.408	8.078	0.472	2.870
2.5	0.321	7.404	0.480	1.313
2.7	0.213	6.763	0.465	0.550
2.9	0.000	6.268	0.444	0.222
3.1	0.000	5.859	0.421	0.099

Table S2. Individual contributions to D-tensor of 1 calculated by CASSCF/NEVPT2.

Multiplicity	Root	*D*	*E*
4	0	0	0
4	1	-29.949	-0.684
4	2	8.174	-9.272
4	3	9.405	9.237
4	4	0.24	-1.274
4	5	0.017	-0.001
4	6	0.029	-0.057
4	7	0.004	-0.009
4	8	-0.013	-0.002
4	9	0	0
2	0	0.529	0.356
2	1	-0.346	-0.359
2	2	-0.004	-0.004
2	3	-0.004	-0.004
2	4	0.117	0.001
2	5	-0.002	-0.002
2	6	4.699	0.149
2	7	-1.808	2.021
2	8	-2.356	-2.324
2	9	-0.059	0.322
2	10	0.305	0
2	11	-0.004	-0.003
2	12	0.373	0.001
2	13	-0.085	0.112
2	14	-0.012	0.017
2	15	-0.134	-0.134
2	16	-0.001	0.031
2	17	-0.005	-0.005
2	18	-0.005	-0.005
2	19	0.127	0.028
2	20	-0.002	-0.003
2	21	0.001	0.014
2	22	-0.133	0.118
2	23	-0.787	-0.787
2	24	-0.399	0.455
2	25	0.58	0.058
2	26	-0.001	-0.001
2	27	-0.002	-0.002
2	28	0	0
2	29	0.198	0.031
2	30	-0.048	0.083
2	31	-0.106	-0.107
2	32	-0.002	-0.002
2	33	-0.009	0.011
2	34	0.001	0
2	35	0	0
2	36	-0.011	-0.011
2	37	-0.011	-0.01
2	38	-0.001	0.016
2	39	0.027	0.002

Table S3. Energy levels (cm-1) of ligand field multiplets in zero magnetic field derived from CASSCF/NEVPT2 calculations for **1**.

0:	0.0000
1:	0.0000
2:	23.5604
3:	23.5604
4:	3087.7150
5:	3087.7150
6:	3195.0710
7:	3195.0710
8:	4715.8572
9:	4715.8572
10:	4822.5659
11:	4822.5659
12:	5967.2453
13:	5967.2453
14:	6029.8930
15:	6029.8930
16:	7276.4912
17:	7276.4912
18:	7372.6205
19:	7372.6205
20:	8049.6123
21:	8049.6123
22:	8162.0525
23:	8162.0525
24:	10852.0787
25:	10852.0787
26:	10872.3037
27:	10872.3037
28:	16947.0917
29:	16947.0917
30:	17335.2832
31:	17335.2832
32:	18388.6677
33:	18388.6677
34:	19009.9128
35:	19009.9128
36:	19296.2182
37:	19296.2182
38:	20046.6569

39:	20046.6569
40:	20114.1896
41:	20114.1896
42:	20263.7498
43:	20263.7498
44:	20372.1791
45:	20372.1791
46:	20455.8885
47:	20455.8885
48:	20885.7132
49:	20885.7132
50:	21065.6457
51:	21065.6457
52:	21223.6365
53:	21223.6365
54:	22123.9809
55:	22123.9809
56:	23063.8059
57:	23063.8059
58:	23350.8728
59:	23350.8728
60:	24144.1906
61:	24144.1906
62:	24464.6112
63:	24464.6112
64:	25454.7898
65:	25454.7898
66:	25805.2320
67:	25805.2320
68:	26069.1511
69:	26069.1511
70:	26817.0420
71:	26817.0420
72:	27361.7543
73:	27361.7543
74:	28140.4443
75:	28140.4443
76:	28395.7822
77:	28395.7822
78:	28623.0659
79:	28623.0659

80:	29091.4119
81:	29091.4119
82:	29813.2046
83:	29813.2046
84:	30155.1438
85:	30155.1438
86:	30640.6275
87:	30640.6275
88:	31006.7925
89:	31006.7925
90:	31612.1961
91:	31612.1961
92:	33389.8795
93:	33389.8795
94:	33801.2081
95:	33801.2081
96:	40017.3128
97:	40017.3128
98:	40510.5463
99:	40510.5463
100:	40926.6559
101:	40926.6559
102:	41233.5456
103:	41233.5456
104:	42117.4383
105:	42117.4383
106:	42398.9642
107:	42398.9642
108:	42706.9395
109:	42706.9395
110:	59646.0935
111:	59646.0935
112:	61068.6903
113:	61068.6903
114:	61391.8453
115:	61391.8453
116:	62199.3130
117:	62199.3130
118:	62429.2287
119:	62429.2287
